# A clinical nursing rotation transforms medical students’ interprofessional attitudes

**DOI:** 10.1371/journal.pone.0197161

**Published:** 2018-05-24

**Authors:** Katrina Butterworth, Rashmi Rajupadhya, Rajesh Gongal, Terra Manca, Shelley Ross, Darren Nichols

**Affiliations:** 1 Department of General Practice, Patan Academy of Health Sciences, Lalitpur, Nepal; 2 School of Nursing, Patan Academy of Health Sciences, Lalitpur, Nepal; 3 Department of General Surgery, Patan Academy of Health Sciences, Lalitpur, Nepal; 4 Canadian Center for Vaccinology, Dalhousie University, Halifax, Canada; 5 Department of Family Medicine, University of Alberta, Edmonton, Canada; 6 Department of Family Medicine, University of Alberta, Edmonton, Canada; University of Newcastle, AUSTRALIA

## Abstract

This study explores the extent to which a one-week nursing rotation for medical students changed the interprofessional attitudes of the participating nurses and students. Third-year medical students worked with nurses before starting clinical rotations. Pre- and post-experience surveys assessing perceptions of mutual respect, nurse-doctor roles, and interprofessional communication and teamwork were given to 55 nurses and 57 students. The surveys consisted of qualitative questions and a Likert scale questionnaire that was analyzed using qualitative and quantitative content analyses. The response rate was 51/57 (89%) students and 44/55 (80%) nurse preceptors. Nurses reported that students met nurses’ expectations by displaying responsibility, respect, effective communication, and an understanding of nursing roles. Medical students’ narratives demonstrated two significant changes. First, their views of nurses changed from that of physician helpers to that of collaborative patient-centred professionals. Second, they began defining nursing not by its tasks, but as a caring- and communication-centred profession. Responses to Likert-scaled questions showed significant differences corresponding to changes described in the narrative. A one-week immersive clinical nursing rotation for medical students was a transformative way of learning interprofessional competencies. Learning in an authentic workplace during a clinical rotation engendered mutual respect between nurses and future doctors. Students’ view of the role of nurses changed from nurses working for doctors with patients, to working with doctors for patients.

## Introduction

Healthy interprofessional relationships are vital for effective team-based patient care, for reducing medical errors, and for improving patient outcomes [1–4]. When physician-nurse relationships are poor, they adversely affect patients, cause job dissatisfaction, and even worsen the personal health of nurses and physicians [5–7]. Interprofessional learning (IPL) holds the promise of improving collaborative practice. IPL or interprofessional education (IPE) occurs when ‘two or more professions associated with health or social care are engaged in learning with, from and about each other’ [8].

The 2015 Institute of Medicine (IOM) report on IPE acknowledged the variety of IPL that occurs between a myriad of health and social care professionals and students in a variety of clinical, classroom, and community settings [9]. Despite, or perhaps because of, this heterogeneity in the delivery of IPL, the IOM highlighted the importance of measuring the impact of IPL at the level of learning outcomes, and the level of patient and system outcomes. So far there is a lack evidence for IPL, and what exists has seldom demonstrated beneficial patient- and system-level outcomes [10–12].

In its report, the IOM suggests using a modified Kirkpatrick framework for measuring the impact of IPL on students and professionals. The IOM called for research into the modification of attitudes, perceptions, and actual behavioural changes between interprofessional groups [13,14]. Systematic reviews of IPL and collaborative practice suggests that IPL is enjoyed by learners, who are enabled to learn the knowledge and skills necessary for collaborative working. However, IPL is not as effective at positively influencing attitudes and perceptions towards other team members, despite this being a clear need in physician-nurse collaboration [15,16].

At the undergraduate level, choosing precisely what type of IPL to do, with whom, and when, is critical [17]. Current literature suggests that the delivery of undergraduate IPE that is done between the domains of medicine and nursing occurs most commonly between students in non-clinical settings [18–22]. Medical students and nursing students often undertake IPL in the classroom setting, but students seldom work with experienced practitioners of the other discipline.

In the past decade, however, robust IPL in the clinical workplace between practicing nurses and medical students has been increasingly reported. This aligns with the evidence that authentic clinical learning opportunities are most beneficial [15]. To date, there remain very few published reports of IPL programs where medical students are taught nursing fundamentals in the clinical workplace by nurses [23–28]. The results of four of these brief 4-hour to 16-hour nurse-shadowing experiences are encouraging, with students reporting an increased knowledge of, and respect for, nursing roles [23–25]. A more extensive 4-week nursing rotation provided a powerful learning experience for first-year Dutch students [28]. This study demonstrated favourable changes in student attitudes towards nurses and their roles, and supports appropriately early clinical exposures for medical students working with nurses. This type of in-depth analysis of attitudinal change is precisely what the Institute of Medicine (IOM) called for in its 2015 report on the evaluation of IPL experiences.

To follow on the IOM report suggestions, we developed a nurse-shadowing rotation for junior medical students in South Asia. This study took place at the Patan Academy of Health Sciences (PAHS) in Nepal. PAHS was founded in 2009 with the socially accountable mission of graduating medical students from all socioeconomic backgrounds with broad competencies including compassion, empathy, professional respect and responsibility, leadership and teamwork. Prior to this study, PAHS faculty held workshops on interprofessional communication skills where the key concerns expressed by nurses were identified as the lack of respect and poor attitudes of doctors towards nurses. The strong hierarchy amongst health professionals often left nurses feeling disregarded, poorly understood, or held in low esteem by their physician colleagues. The nurse-shadowing rotation was designed as a transformative learning experience in which students could be safely uncomfortable in a real-world setting. The goal was to improve students’ understanding of, and respect for, the role of nurses on a healthcare team. The aim of this study was to describe junior medical students’ attitudes towards nurses and collaborative practice before and after a clinical nursing rotation.

## Methods

### Setting

To address the issue of interprofessional respect and understanding, PAHS faculty designed and implemented a one-week nursing rotation for all third-year medical students before they began clinical clerkship. As part of the preparation for the rotation, the director of nursing (RR) and the lead physician (KB) met with the senior nurses in the hospital to discuss what the objectives should be. Each ward’s head nurse was asked to discuss their desired objectives for the medical students. This was an essential step in engaging the nurses. An agreed set of objectives for the rotation, incorporating both technical and non-technical skills, and several attitudinal objectives was produced (Table 1). There was no specific didactic curriculum. The shadow-rotation was left as broadly experiential for two reasons. First, it reduced the workload on the nurses who integrated the students into their daily workflow without needing to attend to extra classroom teaching. Second, it allowed for an educational experience promoting transformative learning, where one’s worldview changes as a result of real-life problem solving and reflection in a supportive, but significantly unfamiliar and uncomfortable environment [29]. The overarching learning objective of this rotation was for students to understand the essential role that nurses play, thereby improving their attitudes and behaviours when working with nurses.

**Table 1:** Nurse-derived rotation objectives (Objectives in bold are the non-technical objectives).

Domain	Objectives: By the end of this rotation the student will be able to:
Knowledge	**Discuss the role of the nurses on the wards, particularly bedside nursing care.** **Discuss the role of nurses-patient communication.** **Discuss holistic nursing care.** Explain infection control measures on the ward. Explain the nurses’ record keeping system. Explain how to administer IV drugs safely. Explain how to administer blood products safely. Discuss the rules and regulations of the hospital.
Skills	**Communicate effectively with patients and their visitors.** **Communicate effectively with the nursing team and other staff (eg. housekeeping)** **Deliver simple health education** Demonstrate good routine care of the patient (washing, feeding, clothing). Demonstrate safe patient handling/transferring. Demonstrate good hand washing. Demonstrate IV line care for infection control. Demonstrate proper disposal of waste products. Demonstrate proper use of equipment, including replacement after use
Attitudes	**Demonstrate punctuality.** **Demonstrate respect towards the nursing team.**

In 2012, all fifty-seven students of the third-year class were assigned by convenience to one of five hospital wards: pediatrics, internal medicine, general surgery, obstetrics and gynecology, and emergency. There they worked for one week as part of the nursing team, both observing and assisting in nursing duties. The nurses supervised one or two students at a time and had no other learners from other disciplines.

### Study design

This was a pre- and post-intervention survey designed to explore the impact of the nursing rotation on medical students' attitudes towards nurses and knowledge of nursing roles. Following a literature search and consultation with an educationalist (SR), we created a specific survey tool to answer the key research questions. The researchers held serial discussions about survey question content and usability to refine the face validity of the questions. Content validity was confirmed by our experts who reviewed the literature and consensus discussion (SR, KB, DN, TM) led to the final design of the pre- and post-questionnaires for nurses and students.

The nurses’ questionnaire was intended to assess their current experience of doctor-nurse relationships and their expectations of students, using a four-point anchored Likert scale. In addition, we expanded the questionnaire with written narrative to explore the impact of this experience, asking the nurses open-ended questions such as: ‘What changes do you want this rotation to make in your communication, interaction or teamwork with these students?’, ‘What was the most valuable thing the students learned from this rotation?’ and ‘How will this rotation change your communication, interaction or teamwork with these students in the future?’. A similar pre- and post- questionnaire was designed for medical students, to assess their attitudes towards interprofessional relationships, using a similar Likert scale. As with the nurses, the student survey was expanded with written narrative to explore students’ experience and the impact of the rotation with questions such as: ‘What is the nurses’ role on the ward?’, ‘What was the most valuable thing you learned from this rotation?’ and ‘How will this rotation change your communication, interaction or teamwork with the nurses in future?’.

### Participants

All 57 third-year medical students (32 female) and all of their 55 supervising nurses (all female) were invited to participate in this survey. This was the first opportunity nurses had to work with medical students in our new medical school. Informed consent of all participants was obtained, and their participation in the study remained confidential. All survey responses were coded for anonymity, and at no time did any of the students’ assessors, nor any of the nurses’ supervisors, have any access to identifiable survey data. The research ethics board of the Patan Academy of Health Sciences granted approval for this educational program evaluation.

### Procedure

All nurses working on the wards and all medical students received questionnaires before the rotation, and again within one week of completing the rotation. The coded surveys allowed linking of pre- and post- data for individuals. The linking codes were known by only one of the researchers (TM).

### Data analysis

Survey data from Likert-scaled questions was analyzed using 2-tailed T-tests. Answers to the open-ended written questions were analyzed by one research assistant (TM) who worked with two of the physicians (DN, KB) to complete the content analysis. Themes were identified and pre-post comparison was carried out independently by three authors (TM, SR, DN). These three authors then met and came to consensus about the presence and significance of the themes evident in the students’ and nurses’ writings.

## Results

Response rates for the questionnaires were 89% (51/57) for students and 80% (44/55) for nurse preceptors. Following the rotation, nurses were more likely to “strongly agree” with 2 statements, 2 and 11 (Table 2). Statement 2 indicated that nurses felt increasingly respected by the students. Statement 11 suggested an ongoing desire for doctors to better understand nursing duties. The nurses were less likely to affirm that students were knowledgeable after the rotation.

**Table 2:** Pre- and post nurse survey questions.

	Nurse Survey Questions (pre and post)	Pre	Post	Pre-post mean Difference (a positive number indicates stronger agreement)	Significance (2-tailed)^a^
*1*.	Doctors and nurses generally work well together.	1.67	1.74	-.077	.373
*2*.	I feel respected by the doctors.	2.26	1.79	.462	.000
*3*.	Nurses are good at listening to the doctors.	1.62	1.49	.128	.201
*4*.	Nursing duties are important to patients’ health.	1.13	1.21	-.077	.262
*5*.	Doctors respect the nurses.	2.10	2.13	-.026	.838
*6*.	Nurses do their job well.	1.33	1.44	-.103	.253
*7*.	Nurses respect the doctors.	1.64	1.56	.077	.446
*8*.	Doctors are good at listening to the nurses.	2.18	2.15	.026	.711
*9*.	Doctors’ care is more important than nursing care.	3.08	2.97	.103	.324
*10*.	Doctors have a difficult job.	2.13	2.10	.026	.767
*11*.	Doctors should know how to perform nursing duties.	2.24	1.79	.447	.008
*12*.	During this rotation, I expect the students will helpful/the students were helpful.	1.79	1.85	-.051	.623
*13*.	During this rotation, I expect the students will be knowledgeable/the students were knowledgeable.	1.62	1.85	-.231	.037
*14*.	During this rotation, I expect the students will be approachable/the students were approachable.	1.72	1.87	-.154	.135

^a^4-point Likert anchors (1 = Strongly agree, 2 = Agree, 3 = Disagree, 4 = Strongly Disagree) were analyzed with a 2-tailed T-test.

Students were more likely to “strongly agree” with questions 2, 3, 4, 6, and 7 after the rotation (Table 3). All of these statements suggest that students felt both increasingly respected and were increasingly respectful of nurses and their roles.

**Table 3:** Pre- and post student survey questions.

	Student Survey (pre and post)	Pre	Post	Pre-post mean Difference (a positive number indicates stronger agreement)	Significance (2-tailed)^a^
*1*.	Doctors and nurses generally work well together.	1.74	1.64	.100	.255
*2*.	I feel respected by the nurses.	1.90	1.56	.340	.001
*3*.	Nurses are good at listening to the doctors.	2.00	1.59	.408	.000
*4*.	Nursing duties are important to patients’ health.	1.16	1.04	.120	.032
*5*.	Doctors respect the nurses.	1.98	1.90	.080	.399
*6*.	Nurses do their job well.	1.84	1.64	.200	.017
*7*.	Nurses respect the doctors.	1.94	1.70	.240	.002
*8*.	Doctors are good at listening to the nurses.	2.14	2.06	.080	.438
*9*.	Doctors’ care is more important than nursing care.	2.80	2.70	.500	.225
*10*.	Nursing is a difficult job.	1.62	1.50	.120	.159
*11*.	Doctors should know how to perform nursing duties.	1.48	1.48	.000	1.000
*12*.	During this rotation, I expect the nurses to be helpful (the nurses were helpful)	1.32	1.18	.140	.070
*13*.	During this rotation, I expect the nurses to be knowledgeable (the nurses were knowledgeable)	1.46	1.46	.000	1.000
*14*.	During this rotation, I expect the nurses to be approachable (the nurses were approachable)	1.42	1.32	.100	.280

^a^4-point Likert anchors (1 = Strongly agree, 2 = Agree, 3 = Disagree, 4 = Strongly Disagree) were analyzed with a 2-tailed T-test.

Both nurses and students wrote extensive responses to the open-ended questions. A thematic analysis of the written content told a more complex and complete story. Responses were analysed together, rather than question-by-question, as there was significant overlap in the themes expressed. The themes are presented below.

### Narrative statements

#### Nurses’ perceptions before and after the rotation

Prior to the rotation, the nurses hoped for three main outcomes: that the students be professionally responsible, respectful, and good communicators. The post-rotation narratives give depth to how these three areas were manifested.

First, nurses felt the students were professional—which expressed itself in their responsibility to the patients, to learning, and to the team.

Nurses stated the students learned to provide “nursing care for patient(s) according to their need, counseling to patients and health education” (Nurse 22). Their “attachment to patient(s) is the main theme they learn from this rotation” (Nurse 33) as was their “development of confidence” (Nurse 28). Students’ enthusiasm for learning was echoed by many nurses.

Second, the nurses identified respect as a distinct quality. This was expressed in how students learned “how much our nursing job is valuable” (Nurse 34), “the importance of (the) nursing role in the patient care” (Nurse 52) and learned to “develop good interpersonal relationships which will help to develop their sense of team spirit and positive attitude toward nurses (Nurse 3). This left nurses hopeful that “they will be nice to us in coming future when they'll really become doctors” (Nurse 17).

Third, improved communication manifested in several ways. The “importance of recording and reporting and documentation” (Nurse 52) was noted in “vital signs recording and reporting” (Nurse 13), and “proper handover and takeover of patients” (Nurse 55).

However, the relational aspects of communication–both colleague- and patient-centeredness–were dominant. “The most valuable thing the students learned from rotation is the relationship between doctor and patient and staffs” (Nurse 17). “It will help to build (the) trust of the staff and work as a team member to provide efficient and quality care” (Nurse 7). Nurses repeatedly noted that students demonstrated “the capacity… to maintain interpersonal relationships with staffs, patients and visitors” (Nurse 9), “total patient care in (a) real situation and how to communicate, interact and work as (a) team member in teamwork” (Nurse 42).

Overall only four nurses noted unprofessional behaviours in the students and these all related to either failing in responsibility to the patient or the team. “Some of them feel ashamed and hesitate in doing minor jobs like bed making, position change but they should know it means great (things) in well-being and (in the) progress of patient” (Nurse 17). Several comments suggested that the nurses felt empowered to address issues of unprofessionalism with their medical students. “The main thing is that if any (poor) behaviour is seen then we can consult with each other rather than side talk” (Nurse 33). This refers to the common practice of gossiping and grumbling about poor behaviour without actually addressing it directly.

A final theme that arose in the written comments described the nurses’ roles as teachers. None of the nurses had ever taught medical students before, and there was minimal faculty development prior to this rotation. In this context only a minority of nurses expressed confidence in teaching. “This rotation provide(s) us a chance to share our ideas, knowledge and experience with each other as well as increase our learning habits, so helped us in good communication, interaction and work as good team creating a good working environment” (Nurse 42). “Sometimes student behaviour may change but we have (the) power to handle it” (Nurse 37).

#### Students’ perceptions before and after the rotation

The summary of the students’ view of nursing roles could perhaps be described as follows: nurses went from working for the doctors and with the patient, to with the doctors and for the patients. The student perception of the role of nurses underwent two large shifts: nurses went from being physician helpers to having independent professional roles; and they went from being task-oriented to patient- and communication-oriented.

The first change in student perception was clear. Before the rotation, nurses were described by a majority of students as being subservient to, and dependent on, doctors. While some students described nurses’ “teamwork with doctors” (Student 17), which “complement(s) doctors’ care” (Student 21), most did not. Nurses were clearly perceived to “assist,” “report to,” “inform,” “help” “discuss with,” “make things ready for,” “follow,” and “call” the doctor (multiple students). They are to “do whatever the doctor directs” (Student 34). Even positive statements that nurses “provide care to the patient…” is only “… as instructed by the doctors” (Student 22).

After shadowing the nurses, the majority of students commented on the independent roles of nurses, and their collaborative management of patients. Nurses are the only ones “there for the patient 24/7” (Student 7), and are “as important as, at times much more important, than doctors” (Student 20). The importance of nursing care was highlighted specifically as: “integral”, “holistic”, “vital”, and “helping in every difficulty” as the nurses pursued “spiritual and emotional care”, “team work”, “good communication with a cheerful face” (multiple students). Witnessing the empathy of nurses who were “feeling the pain of the patients by themselves” (Student 27) prompted students to “know that the role of nurses is much greater in caring and curing of the patients” (Student 48).

The second evolution in students’ view of their nursing colleagues appears to be one that puts the role of communication ahead of the technical tasks of nursing. Prior to the rotation students understood that nursing duties included “comforting” and “educating” the patients as well as “managing beds, giving medications”, “sending investigations, collecting reports”, “maintaining hygiene” and “measuring vitals”. The balance of comments shifted after the rotation. Following their shadow experience, students spoke more often, and with greater depth about non-technical nursing skills. The “job of nurses” in the students’ eyes became much more than “daily duties” and “technical” tasks. Nurses were seen as “more approachable to patients than doctors” (Student 17). Students learned “ethical concern” (Student 18), and empathy: patients “want to be touched by us and given assurance” (Student 45). The empathy students felt for patients extended to the nurses whom they thought were often overworked.

Respect was not only for patients but also with the whole team who all have “an equal part to play”. As one student put it “I felt respected by almost all the nurses, they were helpful and co-operative in every way” (Student 20). Another noted respect from doctor to nurse: “I actually noted a senior doctor actually asking medical advice of one of the nurses on a procedure” (Student 46). As one student summarized: “(I learned) to work as a team, to understand and internalise the troubles and difficulties faced by the nursing staffs, to provide holistic care to the patients, to better meet the patient's physiological, spiritual, emotional, social needs” (Student 52).

Finally, our surveys gave room for comment on iterative improvement of the rotation. The majority of both nursing and student cohorts requested that the rotation be increased in duration. None requested that it be shorter, nor discontinued. A significant minority requested a clearer orientation to students regarding ward duties, and orientation for nurse preceptors regarding student objectives and expectations. Finally, some nurses who found teaching while they were working to be difficult requested dedicated teachers for the students. Many students noted that nurses were hesitant to provide formative feedback.

## Discussion

The findings of this study are encouraging: students and nurses each demonstrated significant changes in their attitudes towards the other professional group following a simple immersive rotation. This is important considering the attention given to defining IPL, and the challenges experienced in best achieving it [14,30]. This study adds to the small body of IPL studies in which students of medicine learn while working with nurses in a clinical setting. While we did not attempt to demonstrate benefit to patient outcomes, nor to objectively assess discrete behaviours, it is clear that this immersive nurse-shadowing rotation for medical students changed the attitudes that are key to enhancing collaboration. Care does need to be taken in interpreting the data of this study: changes in attitudes do not necessarily lead to changes to behaviours nor to patient outcomes. And although the attitudinal changes that were described by participants showed some significant change, the numerical data suggests that the change was often a confirmatory one–nurses and students who, for example, agreed that they felt respected initially, simply agreed more strongly at the end of the rotation. Nonetheless, collaborative attitudes improved.

For an answer to why this authentic workplace IPL experience may have improved understanding and respect, we looked to transformative learning theory. According to O’Sullivan, “transformative learning involves experiencing a deep, structured shift in the basic premises of thought, feelings, and actions. It is a shift in consciousness that dramatically and irreversibly alters our way of being in the world. Such a shift involves our understanding of ourselves and our self-locations; our relationships with other humans and with the natural world; our understanding of relations of power in interlocking structures of class, race and gender, our body awareness, our visions of alternative approaches to living; and our sense of possibilities for social justice and peace and personal joy” [31]. Transformative learning occurs in response to a challenge to one’s world view. Acute personal crises, intercultural experiences, and challenging dialog can all catalyze transformation. This type of learning is further fostered by real-life problem solving, critical reflection, and a flat student-teacher gradient where the teacher is on an equal footing to the student.

Many of these factors are present in our setting at the Patan Academy of Health Sciences. In many ways, this rotation was an intercultural immersion for the medical students. And while the student-teacher gradient was not necessarily flat, it was of a low gradient. Possibly because both medical students and nurses share a position below physicians in the hierarchy of the hospital, there was a comfort each with the other. Nurses had a unique opportunity to teach and influence future doctors; and students got to learn from professionals who make up the team they may ultimately be asked to lead.

The results of this study may encourage changes to how IPL is best delivered. Indeed, we managed to overcome many of the barriers believed to be obstacles to effective IPE in both developed and developing countries [32]. Clinically-based interprofessional learning opportunities are preferable to classroom-based events because they allow students’ learning to be rooted in a relevant, everyday context [33]. The context of learning is critical: learning occurs through the interaction of individuals, their behaviour, and the environment. An authentic context significantly impacts on the ways students perceive their learning experiences [15,34].

The timing of IPE may be critical [35]. Ideally, students and faculty from different disciplines should be confident in sharing from their own knowledge base, while able to listen to, respect, and learn from the knowledge base of others. In our context, where tradition and complex hierarchies lead to significant power differentials between doctors and nurses, we chose to introduce IPE to mid-level medical students on their first significant exposure to clinical work on the wards. In this situation, they were not confident in their own knowledge base, but were also less likely to be firmly embedded in a negative anti-collaborative attitude to practice. Effective collaboration is grounded in the willingness of different professionals to share their specialist knowledge with each other while recognizing and respecting the knowledge of those from other professions [36,37]. Our junior students were ripe for transformative learning with their first major clinical experience being in the unfamiliar world of ward nursing, under the supportive guidance of their senior nursing colleagues.

## Limitations

This study had several limitations. The largest of these is the potential for participants writing down the “right answer” even if it does not reflect actual beliefs or practices. We are not convinced that the anonymity of the surveys was adequate to completely counteract this cultural practice amongst our students and nurses. However, this social desirability bias is likely to have affected both pre- and post-surveys.

Inherent to the methodology, this was a student-centric study that did not focus on the learning of the nurses as new preceptors. The placement of students in heterogeneous environments may make some of the comparisons unequal, and we did not analyze each separate nursing ward to see if there were obvious differences between the settings. Unlike many classroom-based IPE programs, we did not evaluate a common curriculum delivered to all students, but rather the set of individual experiences in the workplace. The next step of this research may be to use objective measures to assess ongoing behaviours of collaborative practice between these nurses and students over time.

## Conclusion

A one-week clinical nursing rotation for medical students was an authentic way of learning interprofessional competencies around collaboration, communication, and professionalism. It enhanced respect for the nursing profession in students and engendered respect for the students by nurses. Even this brief rotation resulted in transformative learning, where students recognized nurses as independent health practitioners within the health care team and identified nurses as holistic patient-centred communicators.

## Supporting information

S1 File Student and nurse pre and post surveys. This file contains the surveys used in the study.
(DOCX)

S2 File Student_nurse surveys pre_post data complete. This file contains the complete compiled study data.
(XLS)
