# Supplementary material for: A clinical nursing rotation transforms medical students’ interprofessional attitudes
Source: PLoS One. 2018 May 24;13(5):e0197161. doi: 10.1371/journal.pone.0197161 (PMC5967832; doi:10.1371/journal.pone.0197161)
Supplement: S1 File — This file contains the surveys used in the study. (DOCX) [file pone.0197161.s001.docx]

Student pre-rotation survey

|  | Question | Strongly Agree | Agree | Disagree | Strongly Disagree |
| --- | --- | --- | --- | --- | --- |
| 1. | Doctors and nurses generally work well together. |  |  |  |  |
| 2. | I feel respected by the nurses. |  |  |  |  |
| 3. | Nurses are good at listening to the doctors. |  |  |  |  |
| 4. | Nursing duties are important to patients’ health. |  |  |  |  |
| 5. | Doctors respect the nurses. |  |  |  |  |
| 6. | Nurses do their job well. |  |  |  |  |
| 7. | Nurses respect the doctors. |  |  |  |  |
| 8. | Doctors are good at listening to the nurses. |  |  |  |  |
| 9. | Doctors’ care is more important than nursing care. |  |  |  |  |
| 10. | Nursing is a difficult job. |  |  |  |  |
| 11. | Doctors should know how to perform nursing duties. |  |  |  |  |
| 12. | During this rotation, I expect the nurses to be helpful |  |  |  |  |
| 13. | During this rotation, I expect the nurses to be knowledgeable |  |  |  |  |
| 14. | During this rotation, I expect the nurses to be approachable |  |  |  |  |

Open-ended questions for students pre-rotation:

1. What is the nurses’ role on the ward?

2. How do you think this rotation will change your communication/interaction/teamwork with the nurses in future?

Student post-rotation survey

|  | Question | Strongly Agree | Agree | Disagree | Strongly Disagree |
| --- | --- | --- | --- | --- | --- |
| 1. | Doctors and nurses generally work well together. |  |  |  |  |
| 2. | I feel respected by the nurses. |  |  |  |  |
| 3. | Nurses are good at listening to the doctors. |  |  |  |  |
| 4. | Nursing duties are important to patients’ health. |  |  |  |  |
| 5. | Doctors respect the nurses. |  |  |  |  |
| 6. | Nurses do their job well. |  |  |  |  |
| 7. | Nurses respect the doctors. |  |  |  |  |
| 8. | Doctors are good at listening to the nurses. |  |  |  |  |
| 9. | Doctors’ care is more important than nursing care. |  |  |  |  |
| 10. | Nursing is a difficult job. |  |  |  |  |
| 11. | Doctors should know how to perform nursing duties. |  |  |  |  |
| 12. | During this rotation, the nurses were helpful |  |  |  |  |
| 13. | During this rotation, the nurses were knowledgeable |  |  |  |  |
| 14. | During this rotation, the nurses were approachable |  |  |  |  |

Open-ended questions for students post-rotation:

1. What was the most valuable thing you learned from this rotation?

2. How will this rotation change your communication, interaction or teamwork with the nurses in future?

3. Were you concerned about any behaviours by nurses, students, or doctors on this rotation? How could this be fixed?

4. Do you have any suggestions to improve this rotation?

5. Do you have any other comments about this rotation?

Nurse pre-rotation survey questions

|  | Questions | Strongly Agree | Agree | Disagree | Strongly Disagree |
| --- | --- | --- | --- | --- | --- |
| 1. | Doctors and nurses generally work well together. |  |  |  |  |
| 2. | I feel respected by the doctors. |  |  |  |  |
| 3. | Nurses are good at listening to the doctors. |  |  |  |  |
| 4. | Nursing duties are important to patients’ health. |  |  |  |  |
| 5. | Doctors respect the nurses. |  |  |  |  |
| 6. | Nurses do their job well. |  |  |  |  |
| 7. | Nurses respect the doctors. |  |  |  |  |
| 8. | Doctors are good at listening to the nurses. |  |  |  |  |
| 9. | Doctors’ care is more important than nursing care. |  |  |  |  |
| 10. | Doctors have a difficult job. |  |  |  |  |
| 11. | Doctors should know how to perform nursing duties. |  |  |  |  |
| 12. | During this rotation, I expect the students will helpful. |  |  |  |  |
| 13. | During this rotation, I expect the students will be knowledgeable. |  |  |  |  |
| 14. | During this rotation, I expect the students will be approachable. |  |  |  |  |

Open-ended questions for nurses pre-rotation:

1. What was the most important thing you want the students to learn from this rotation?

2. What changes do you want this rotation to make in your communication, interaction or teamwork with these students?

Nurse post-rotation survey questions

|  | Questions | Strongly Agree | Agree | Disagree | Strongly Disagree |
| --- | --- | --- | --- | --- | --- |
| 1. | Doctors and nurses generally work well together. |  |  |  |  |
| 2. | I feel respected by the doctors. |  |  |  |  |
| 3. | Nurses are good at listening to the doctors. |  |  |  |  |
| 4. | Nursing duties are important to patients’ health. |  |  |  |  |
| 5. | Doctors respect the nurses. |  |  |  |  |
| 6. | Nurses do their job well. |  |  |  |  |
| 7. | Nurses respect the doctors. |  |  |  |  |
| 8. | Doctors are good at listening to the nurses. |  |  |  |  |
| 9. | Doctors’ care is more important than nursing care. |  |  |  |  |
| 10. | Doctors have a difficult job. |  |  |  |  |
| 11. | Doctors should know how to perform nursing duties. |  |  |  |  |
| 12. | During this rotation, the students were helpful. |  |  |  |  |
| 13. | During this rotation, the students were knowledgeable. |  |  |  |  |
| 14. | During this rotation, the students were approachable. |  |  |  |  |

Open-ended questions for nurses post-rotation:

1. What was the most valuable thing the students learned from this rotation?

2. How will this rotation change your communication, interaction or teamwork with these students in the future?

3. Were you concerned about any behaviours by nurses, students, or doctors on this rotation? How could this be fixed?

4. Do you have any suggestions to improve this rotation?

5. Do you have any other comments about this rotation?
